# Knowledge on cervical cancer, attitude toward its screening, and associated factors among reproductive age women in Metu Town, Ilu Aba Bor, South West Ethiopia, 2018: community‐based cross‐sectional study

**DOI:** 10.1002/cnr2.1382

**Published:** 2021-05-02

**Authors:** Keneni Chali, Dereje Oljira, Tesfaye Sileshi, Tilahun Mekonnen

**Affiliations:** ^1^ Ilu Aba Bor Zonal Health Department Oromia Regional Health Bureau Metu Ethiopia; ^2^ Faculty of Public Health and Medical Sciences Mettu University Metu Ethiopia; ^3^ Faculty of Health Science, Department of Nursing Mizan Tepi University Tepi Ethiopia

**Keywords:** attitude, cervical cancer, Ethiopia, knowledge

## Abstract

**Background:**

Cervical cancer is one of the major public health problems worldwide. Lack of awareness and unavailability of screening services are the major factors that contribute to the problem of cervical cancer in Ethiopia. The community‐based study conducted regarding the knowledge and attitude toward cervical cancer among women of reproductive age group is not enough to indicate the problem.

**Aim:**

To assess the knowledge on cervical cancer, attitude toward its screening, and associated factors among women of reproductive age.

**Methods:**

A community‐based cross‐sectional study with a mixed approach method was conducted from April to May 2018. The sample size calculated for this study was 420. A systematic random sampling technique was used to select study participants. A binary logistic regression model was used to determine the association between the covariate and the dependent variable.

**Result:**

Of all participants, 31% have good knowledge of cervical cancer, and 57.8% have a positive attitude toward cervical cancer screening. In a multivariable analysis, educational status, occupation, visiting health facilities, and parity were significantly associated with knowledge and attitude toward cervical cancer screening.

**Conclusion:**

This study suggests increasing women's awareness, health education on cervical cancer in the community, and health institutions should be strengthened.

**Patient and public contribution:**

Female health workers were involved in the data collection process. Educated women and women who are community health leaders were involved as Interviewees for the qualitative part of the study. However, they have no direct contributions to authorship.

## 1 INTRODUCTION

Cervical cancer is an abnormal growth of cells in the lining of the cervix. The most common cause of cervical cancer is the human papillomavirus (HPV) infection. Cervical cancer is one of the major public health problems of women worldwide.^1^, ^2^ Globally, cervical cancer is the leading cause of cancer death in sub‐Saharan Africa, Latin America, and many Asian countries.^3^ It is the fourth most common cancer with an estimation of 570,000 cases and 311,000 deaths in 2018 and the most diagnosed cancer and the leading cause of death in 42 countries. Regionally, the highest incidence of cervical cancer is seen in Africa, with an increased rate in southern, Eastern, and Western Africa, respectively.^4^ The incidence of cervical cancer varies among countries of the world. The incidence and death rate in East Africa and West Africa is five times higher than in North Africa.^5^, ^6^The incidence is increasing in African countries. However, knowledge and awareness about cervical cancer are very poor and mortality due to cervical cancer is high. In addition to this weak health system, lack of health care infrastructure, lack of screening service, and lack of trained health care workers exacerbate the cervical cancer problem.^4^, ^7^ The majority of women report that they want to be screened, that is positive attitude, for cervical cancer; however, they complain about lack of screening services.^8^, ^9^, ^10^, ^11^In Ethiopia, cervical cancer is the second most common cancer in women. According to the estimation of 2012, annually 7,095 cervical cancer cases are diagnosed and 4,732 cervical cancer death is occurred in Ethiopia.^12^ Based on 2013 data from the Addis Ababa cancer registry, cervical cancer accounts for about 14.3% of all cancer cases.^13^ A higher proportion of cervical cancer is recorded in Addis Ababa, Oromia, and Amhara regions (32.98%, 30.11%, and 19.72%, respectively).^14^

In Ethiopia, lack of awareness, competing health interests (like Malaria, TB, HIV, etc.), unavailability of cervical cancer screening services, and treatment are the major factors that contribute to the problem of cervical cancer. Recently, few studies showed that lack of awareness is the major factor that influences the knowledge concerning cervical cancer and its screening.^15^ Also, there is a gap of information on factors associated with the knowledge and attitude of women toward cervical cancer.

The objective of this study was to assess the knowledge and attitude of women on cervical cancer and what factors can influence this knowledge in Mettu town, Ethiopia. The findings of this study provide additional information about the level of knowledge and attitude among women in the community on cervical cancer that can help program planners and health educators to design targeted and tailored strategies to increase cervical cancer knowledge and potentially increase cervical cancer screening uptake in Ethiopian.

## 2 METHODS AND MATERIALS

### 2.1 Study design and setting

A community‐based cross‐sectional study design with mixed approach (Quantitative and Qualitative) methods was used to assess knowledge about cervical cancer and attitude toward its screening among women of reproductive age in Mettu town Ilu Aba Bor Zone, Oromia Region, Ethiopia from April to May 2018. The town is the capital of the Ilu Aba Bor Zone, which is located 600 km away from Addis Ababa and located in the southwestern part of the Oromia region. Administratively, Mettu town is subdivided into three Kebeles (unpublished Mettu town administration reportort of 2017). A kebele is the smallest administrative unit in Ethiopia Data S1.

### 2.2 Study participants

The source population was women of reproductive age (15 to 49) years in Mettu town and the study population was all sampled women of reproductive age who live in the selected house holds.

### 2.3 Sample size and sampling techniques

#### 2.3.1 Quantitative part

The required sample size for the quantitative study was estimated by using single population formula (*n* = (Z *α*/2)^2^ *P* (1 − *P*)/*d* ^2^) based on the following assumptions; the proportion of good knowledge of cervical cancer (53.7 %) from the study conducted in Hossana town^16^ and at 95% Confidence level, 5% margin of error and 10% non‐response rate. Thus, the final sample size was 420. A systemic random sampling method was used to selects households and women for interviews.

#### 2.3.2 Qualitative part

A purposive sampling technique was used. A total of 15 key informants were selected by the authors based on their age, educational level, and responsibilities, and experience. Thus, educated women (secondary and above), women above 25 years of age, employed women, and women who are community health leaders were selected.

#### 2.3.3 Data collection tools and procedures

A pre‐tested structured interviewer‐administered questionnaire was used to collect the quantitative data from the study participants. The questionnaire was designed based on the study objectives and adapted from related different survey tools and literature.^10^, ^16^, ^17^, ^18^ It was first prepared in English and was translated to the local language (Afan Oromo) and back‐translated to English to check for its consistency. All data collectors and supervisors were well trained on the data collection procedure. Day‐to‐day supervision was carried out for the entire length of the data collection.For the qualitative part, an in‐depth interview (IDI) with a semi‐structured interview was conducted. Selected key informants were asked about cervical cancer, its risk factors, its prevention as well as their attitude toward cervical cancer screening.

#### 2.3.4 Data processing and analysis

For the quantitative part, the collected data were checked, coded, and entered into Epi‐Data version 3.1 and then exported to SPSS 20 for analysis. Frequencies and cross‐tabulations were used to summarize descriptive statistics. The associated factors were assessed by the Binary logistic regression model. Odds Ratio estimated with 95% CI to show the strength of association and *P*‐value *<* .05 was used to declare statistical significance.For the qualitative study, explanatory sequential mixed methods were used. Each IDI was tape‐recorded and note taken. The data, which were recorded with the audiotape, were transcribed verbatim, translated, and coded. After transcribing, similar themes of the qualitative information were arranged by using thematic analysis and were triangulated or explored with the quantitative finding.

### 2.4 Study variables

Knowledge of cervical cancer and attitude toward cervical cancer screening are dependent variables. Whereas, socio‐demographic factors (age, education of a mother, education of husband, marital status, occupation of a mother, occupation of husband), health service‐related factors (ever visit health institution, use modern contraceptive, ever had HIV test, history of STI), obstetrics factors (parity, history of abortion), and socioeconomic factors (monthly income) are independent variables.

### 2.5 Measurement

**Knowledge**: Ten questions regarding knowledge about the cause (risk factors), symptoms, treatment options, and prevention methods of cervical cancer were taken. Those score above and equal to 5(≥50%) was considered as good knowledge and those score below 5(<50%) was considered as poor knowledge.^19^, ^20^ **Attitude**: Was assessed by eight questions on a Likert scale of five scores. These five scores are Strongly Disagree, Disagree, Neutral, Agree, and Strongly Agree. The mean score was calculated to use as a cut point.^21^

## 3 RESULTS

### 3.1 Socio‐demographic characteristics of the participants

A total of 410 women participated in the study making the response rate 97.6 %. The mean age of the participants was 29.08 and ±7.352 standard deviations (95 % CI: 28.34, 29.72). Nearly half of 192(46.8%) of the participants were in the age group 25‐34 years. Among the total participants, 163(39.8%) were protestant followed by orthodox and Muslim religion followers 132(32.2%) and 88(21.5%), respectively.Nearly two‐thirds (65.4%) of the participants were Oromo followed by Amhara (21%) and Gurage (9%). More than two‐thirds of 284(69.3%) of the participants were married and living with their husbands. Nearly one‐third of 125(30.5%) of the participants have attended secondary^9^, ^10^, ^11^, ^12^ school. Concerning the occupation of the participants, 185(45.1%) of the participants are housewives (Table 1).

**TABLE 1:** Socio‐demographic characteristics of participants of women in Mettu town, Southwest Ethiopia, 2018

Variable	Categories	Frequency	Percent
**Age category (*n* = 410)**			
	15‐24	115	28
	25–34	192	46.8
	35‐44	92	22.4
	44‐49	11	2.7
	Mean + SD	29.11 ± 7.389	
**Marital status (*n* = 410)**			
	Single	91	22.2
	Married	288	70.2
	Separate/Divorced	19	4.6
	Widowed	12	2.9
**Educational status (*n* = 410)**			
	Cannot read and write	41	10
	Can read and write	52	12.7
	Primary^1^, ^2^, ^3^, ^4^, ^5^, ^6^, ^7^, ^8^	105	25.6
	Secondary^9^, ^10^, ^11^, ^12^	127	31
	College and above	85	20.7
**Occupation (*n* = 410)**			
	Employed	70	17.1
	Housewife	186	45.4
	Merchant	49	12
	Student	63	15.4
	Daily laborer	42	10.2
**Household income (*n* = 410)**			
	No regular income	44	10.7
	<33 USD	76	18.5
	33‐58 USD	79	19.3
	58‐98 USD	71	17.3
	>98 USD	139	33.9
**Partner education (*n* = 336)**			
	Cannot read and write	6	1.8
	Can read and write	37	11
	Primary^1^, ^2^, ^3^, ^4^, ^5^, ^6^, ^7^, ^8^	72	21.4
	Secondary^9^, ^10^, ^11^, ^12^	109	32.4
	College and above	112	33.3
**Partner Occupation (*n* = 336)**			
	Employed	99	29.6
	Merchant	99	29.6
	Farmer	51	15.2
	Student	19	5.7
	Daily Laborer	59	17.6
	Others^a^	8	2.4

^a^ Pastor (*n* = 1); driver (*n* = 7).

### 3.2 Health service‐related and obstetrics factors

More than three‐fourth (79.5%) of the respondents were ever used modern contraceptives in their lifetimes. More than three‐fourth (79.3%) of the respondents were ever visited health facilities for any service at least once (Table 2).

**TABLE 2:** Health service‐related and obstetrics factors of the respondents of in Mettu town, Southwest Ethiopia, 2018

Variables	Categories	Frequency	Percent
Used modern contraceptive in their lifetime	YES	326	79.5
	NO	84	20.5
Had HIV test in the facilities	YES	316	77.1
	NO	94	22.9
History of STI	YES	35	8.5
	NO	375	91.5
Visited health institution for any service	YES	325	79.3
	NO	85	20.7
History of abortion	YES	31	7.6
	NO	379	92.4
Parity	Nulliparous	102	24.9
	Primiparous	141	34.4
	Multiparous	167	40.7

### 3.3 Knowledge of women about cervical cancer

**FIGURE 1:**
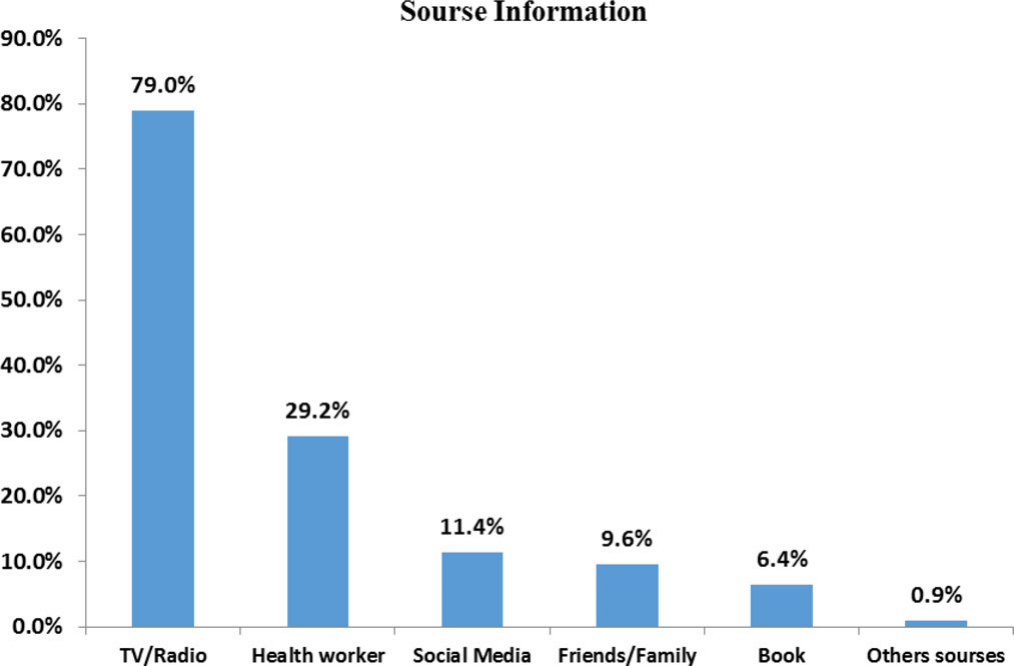
Source of information about cervical cancer (*n* = 219) among respondents in Mettu town, Southwest Ethiopia, 2018

More than half (53.4%) of the participants were ever heard about cervical cancer. Among those who heard about cervical cancer (*n* = 219), 79% heard from TV/Radio and 64(29.2%) of them heard from health workers (Figure 1).Based on the operation definition given, among the total 410 participants, only 127(31%) 95%CI (26.1, 35.1) have good knowledge about cervical cancer; whereas, 283(69%) of the participants have poor knowledge.Nearly one‐fourth (23.2%) of the participants know the risk factors of cervical cancer. The most frequently mentioned risk factors of cervical cancer are early initiation of sexual intercourse 51(53.7%), cigarette smoking 44(46.3%), and having multiple sexual partners 44(46.3%).Finding from IDIs indicate that most of the participants have no adequate knowledge about the risk factors of cervical cancer. The majority of them could not mention any risk factor of cervical cancer. Only some of them mentioned the early initiation of sexual intercourse as a risk factor.One respondent said,

…. “I think mothers are the risk group. Concerning the risk factors, it is difficult for me to explain them. I do not have awareness about the risk factors”About 117(28.5%) of the participants know prevention methods for Cervical Cancer. The most frequently mentioned preventive methods are Avoiding Multiple sexual partners 67(57.3%), avoiding early initiation of sexual intercourse 66(56.4%), Quit Smoking 56 (47.9%), and using condoms 54(46.2%).Finding from In‐depth Interview (IDIs) indicates that the majority of the participants have poor knowledge concerning cervical cancer prevention. They thought that cervical cancer cannot be prevented.

A 33 years respondent said,

…“ Is it preventable? I do not know whether cervical cancer is preventable or not. If it is preventable, women should protect themselves by checking up themselves frequently.”

Another Respondent said,

… Since I haven't screened and counseled, I don't know but people say many things concerning the prevention. They said avoiding plastic materials and different unnecessary foods can prevent cervical cancer.”

### 3.4 Attitudes of participants toward cervical cancer

**FIGURE 2:**
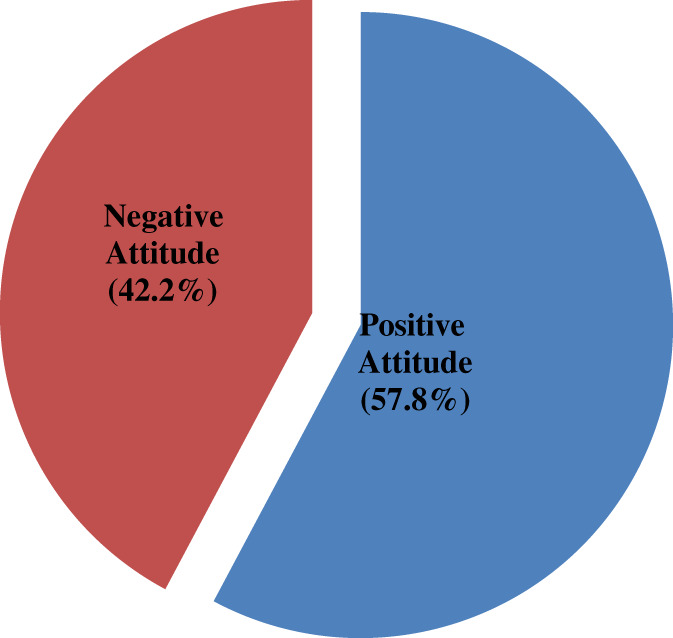
Overall attitude toward cervical cancer among women of reproductive age in Mettu town, 2018

A total of eight questions were put on the Likert scale to assess the attitude of study participants toward cervical cancer screening. The mean score was computed and those who scored mean and above were considered as they have a positive attitude toward cervical screening. Thus, out of 410 participants of this study, 237(57.8%); 95%CI (52.5,63.4) have positive attitudes toward cervical cancer (Figure 2).

Finding from IDIs indicate that the majority of the participants have a positive attitude toward cervical cancer screening. They agreed to be screened frequently, if there is a screening service in health facilities and they said women should be screened for cervical cancer.One 32‐year old woman said

“All of us should be screened. I am informing you that women should be screened frequently. I have a good attitude. I recommend that women should be screened frequently. Early screening is used to detect the disease at an earlier stage. But if she screened at the late stage she cannot cure, so early screening is used for prevention.”

### 3.5 Factors associated with knowledge of cervical cancer

The bivariate analysis showed that socio‐demographic factors, obstetric, and health service‐related factors were seen by bi‐variable logistic regression analysis. Age, marital status, educational status, occupation, partner education, partner occupation, income, parity, and ever visit health institution were significantly associated with knowledge of cervical cancer at *P*‐value ≤ .2. In the multiple logistic regressions education statuses, occupation, ever visited health facility, and parity were found to be a statistically significant association with knowledge of cervical cancer.Women with college and above education were six times more likely to have good knowledge about cervical cancer than women who cannot read and write (AOR: 6.05; 95%CI:1.69, 21.56); and women with secondary^9^, ^10^, ^11^, ^12^ education were more than three times more likely to have good knowledge about cervical cancer than women who cannot read and write (AOR: 3.47; 95% CI: 1.06, 11.35).Employed women were 4.44 times more likely to have good knowledge about cervical cancer than a daily laborer (AOR: 4.44; 95%CI: 1.26, 15.62). Similarly, women who ever visited health institutions for any service were more than two times more likely to have Good knowledge about cervical cancer than women who ever visited health institutions (AOR: 2.39 95% CI: 1.11, 5.17). Additionally, primiparous women were nearly four times more likely to have good knowledge about cervical cancer 3.73 (AOR: 3.73; 95%CI: 1.63, 8.51) (Table 3).

**TABLE 3:** Factors associated with knowledge of cervical cancer among women of age reproductive group in Mettu town, Southwest Ethiopia, 2018

Variables	Knowledge of cervical cancer		Crude OR	Adjusted OR
	Good	Poor		
**Age**				
15–24	34(29.6%)	81(70.4%)	1	1
25–34	70(36.5%)	122(63.5%)	1.37(0.83,2.25)	1.64(0.75,3.55)
35–44	20(21.7%)	72(78.3%)	.66(0.35,1.25)	1.42(0.53,3.83)
≥45	3(27.3%)	8(72.7%)	.89(.22,3.57)	5.35(0.73,39.42)
**Educational status**				
Cannot read and write	4(9.8%)	37(90.2%)	1	1
Able to read and write	15(28.8%)	37(71.2%)	3.75(1.14,12.37)	3.15(0.87,11.34)
Primary^1^, ^2^, ^3^, ^4^, ^5^, ^6^, ^7^, ^8^	20(19%)	85(81%)	2.18(0.69,6.811)	1.74(0.52,5.82)
Secondary^9^, ^10^, ^11^, ^12^	37(29.1%)	90(70.9%)	3.80(1.27,11.43)	**3.47(1.06,11.35)**
College and above	51(60%)	34(40%)	13.87(4.53,42.48)	**6.05(1.69,21.56)**
**Marital status**				
Single	34(37.4%)	57(62.6%)	1	1
Married	80(29.2%)	204(70.8%)	0.69(0.42,1.132)	0.42(0.17,1.03)
Separate/divorced	6(31.6%)	13(68.4%)	0.77(0.26,2.23)	0.52(0.08,2.98)
Widowed	3(25%)	9(75%)	0.56(0.14,2.21)	1.81(0.08,38.85)
**Occupation**				
Employed	42(60%)	28(40%)	7.50(2.93,19.24)	**4.44(1.26,15.62)**
Housewife	47(25.3%)	139(74.7%)	1.69(0.70,4.06)	2.13(0.69,6.48)
Merchant	10(20.4%)	39(79.6%)	1.28(0.44,3.73)	1.26(0.31,5.03)
Student	21(33.3%)	42(66.7%)	2.5(0.95,6.56)	4.03(0.98,16.46)
Daily laborer	7(16.7%)	35(83.3%)	1	1
**Visiting health institution**				
Yes	108(33.3%)	216(66.7%)	1.76(1.01,3.08)	**2.39(1.11,5.17)**
No	19(22.1%)	67(77.9%)	1	
**Parity**				
Nulliparous	24(23.5%)	78(76.5%)	1	1
Primiparous	75(53.2%)	66(46.8%)	3.69(2.10,6.49)	**3.73(1.63, 8.51)**
Multiparous	28(16.8%)	139(83.2%)	.65(0.35,1.21)	0.76(0.33, 1.81)

Bold values is to easily identify the significant variable.

### 3.6 Factors associated with attitude of women toward cervical cancer screening

The bivariate analysis showed that socio‐demographic factors, obstetric, and health service‐related factors were seen by bi‐variable logistic regression analysis. Age, marital status, educational status, occupation, partner education, partner occupation, income, parity, ever visit health institution, and ever used modern contraceptive were significantly associated with attitude toward cervical cancer screening at *P*‐value ≤ 0.2. In the multiple logistic regressions education statuses, and parity has been found a statistically significant association with attitude toward cervical cancer screening.Participants who cannot read and write were 97% times less likely to have a positive attitude toward cervical cancer screening when compared to participants with college and above educational level (AOR: 0.03; 95%CI: 0.01, 0.10). Similarly, participants who were able to read and write were 95% times less likely to have a positive attitude toward cervical cancer screening when compared with college and above educational level (AOR: 0.05; 95% CI: 0.02, 0.15); and women with primary education were 93% times less likely to have a positive attitude toward cervical cancer screening when compared to women with college and above educational status (AOR: 0.07; 95%CI: 0.03, 0.17). In addition to these, multiparous participants were 2.49 times more likely to have a positive attitude toward cervical cancer screening than nulliparous women (AOR: 2.49; 95% CI: 1.09, 5.72) (Table 4).

**TABLE 4:** Factors associated with attitude toward cervical cancer screening among participants in Metu Town, June 2018

Variables	The attitude of Women toward cervical cancer screening		Crude OR	Adjusted OR
	Positive	Negative		
**Age category**				
15–24	67(58.3%)	48(41.7%)	1	1
25–34	112(58.3%)	80(41.7%)	1.00(0.63,1.60)	0.62(0.28,1.37)
35–44	55(59.8%)	37(40.2%)	1.01(0.61,1.86)	1.11(0.42,2.93)
44–49	3(27.3%)	8(72.7%)	0.27(0.68,1.07)	0.74(0.12,4.49)
**Marital status**				
Single	57(62.6%)	34(37.4%)	1	1
Married	161(55.9%)	127(44.1%)	0.76(0.46,1.23)	0.49(0.17,1.41)
Separate/divorced	14(73.7%)	5(26.3%)	1.67(0.55,5.05)	1.04(0.15,7.14)
Widowed	5(41.7%)	7(58.3%)	0.43(0.13,1.45)	0.27(0.01,9.01)
**Educational status**				
Cannot read and write	9(22%)	32(78.0%)	0.05(0.02,0.12)	**0.03(0.01,0.10)**
Can read and write	18(34.6%)	34(65.4%)	0.09(0.04,0.12)	**0.05(0.02,0.15)**
Primary^1^, ^2^, ^3^, ^4^, ^5^, ^6^, ^7^, ^8^	39(37.1%)	66(62.9%)	0.09(0.05,0.20)	**0.07(0.03,0.17)**
Secondary^9^, ^10^, ^11^, ^12^	98(77.2%)	29(22.8%)	0.57(0.26,1.16)	0.790.31,2.01)
College and above	73(85.9%)	12(14.1%)	1	1
**Occupation**				
Employed	55(78.6%)	15(21.4%)	2.75(1.19, 6.35)	0.71(0.19, 2.68)
Housewife	94(50.5%)	92(49.5%)	0.76(0.39,1.51)	0.60(0.22,1.67)
Merchant	28(57.1%)	21(42.9%)	1.0(0.44, 2.30)	0.62(0.18,2.11)
Student	36(57.1%)	27(42.9%)	1.0(0.45, 2.20)	0.21(0.05,0.86)
Daily laborer	24(57.1%)	18(42.9%)	1	1
**Parity**				
Nulliparous	55(53.9%)	47(46.1%)	1	1
Primiparous	90(63.8%)	51(36.2%)	1.51(0.89,2.54)	2.19(0.95,5.07)
Multiparous	92(55.1%)	75(44.9%)	1.04(0.63,1.72)	**2.49(1.09,5.72)**
**Knowledge of cervical cancer**				
Poor knowledge	149(52.7%)	134(47.3)	1	1
Good knowledge	88(69.3%)	39(30.7%)	2.03(1.30,3.16)	1.18(0.59,2.36)

Bold values is to easily identify the significant variable.

## 4 DISCUSSION

This study was a community‐based study conducted to assess the knowledge, attitude, and associated factors among the reproductive age group of Mettu town.This study addressed the knowledge, attitude, and associated factors toward cervical cancer screening. In this study, 53.4% of participants heard about cervical cancer, which is lower than the study conducted in China (85%) and in Dessie town Ethiopia (57.7%).^22^, ^23^ This difference might be due to the gap in the health education system concerning cervical cancer in the current study area.This study showed that only 31% of participants had good knowledge about cervical cancer, which is consistent with the study conducted in Gondar Ethiopia (31%).^17^ The findings from this study showed that overall the knowledge about cervical cancer is higher than the study conducted in Pakistan (23%)^20^ and lower than the study conducted in Uganda (55.4%),^24^ Addis Ababa (37.4%),^25^ and Hossana town Ethiopia (53.7%).^16^ This difference might be due to the difference in awareness creation status concerning cervical cancer in our study area, economic, and socio‐cultural differences of the community. Findings from IDIs indicate that majority of the respondents have poor knowledge concerning cervical cancer prevention. They thought that cervical cancer cannot be prevented. They did not have adequate awareness about prevention methods. Most of the respondents are not aware of the prevention of cervical cancer and those who heard about it do not know its prevention. This may show that the awareness created about cervical cancer prevention in this study area is almost poor.

The finding of this study revealed that participants with college and above education were 6.05 times more likely to have good knowledge about cervical cancer than participants who cannot read and write. Also, participants with secondary education were 3.47 more likely to have good knowledge about cervical cancer than those who cannot read and write. A similar result was published in China, India,^19^, ^22^ and Dessie Town, Ethiopia.^23^ This might be because highly educated women may have many opportunities to get information about cervical cancer.In this study, employment, visiting health institutions, and parity are factors associated with knowledge of cervical cancer. This result is similar to the study conducted in the Democratic Republic of Congo.^26^ This might be because employed women have the chance to get health information through modern technologies.This study revealed that a positive attitude toward cervical cancer screening of the participants is 57.8%. Similar results were published for Finote Selam, North West Ethiopia (58.1%).^21^This result is inconsistent with the study conducted in the Republic of democratic Congo and Dessie Town. The difference might be due to the nature of the population and socio‐cultural difference.^23^, ^26^In this study, educational status and parity were significantly associated with attitude toward cervical cancer screening. This study is consistent with a study conducted in China, India, and Gondor Town, Northwest Ethiopia, and Dessie Town.^17^, ^19^, ^22^, ^23^ This is possible since educated women know the advantage of screening. Parity is also associated with a positive attitude in this study.

## 5 CONCLUSION

This study underlines that overall knowledge about cervical cancer is low. Also, 57.8% of the participants have a positive attitude toward cervical cancer screening. The result of this study showed that educational status, occupation, visiting health facilities, and parity are factors associated with knowledge about cervical cancer. Increasing women's awareness, health education on cervical cancer should be strengthened.

**Abbreviations**
AOR: adjusted odds ratio
CI: confidence interval
HIV: human immunodeficiency virus
HPV: human papilloma virus
IDIs: in‐depth interviews
OR: odds ratio
SD: standard deviation
SPSS: statistical package for social sciences
STI: sexual transmitted infection

## AUTHOR CONTRIBUTIONS

**Keneni Chali:** Conceptualization; data curation; formal analysis; investigation; methodology; software; writing‐original draft. **Dereje Donacho:** Formal analysis; methodology; software; supervision; validation; writing‐review & editing. **Tesfaye Sleshi:** Formal analysis; methodology; supervision; validation; writing‐review & editing. **Tilahun Regassa:** Formal analysis; methodology; software; validation; writing‐review & editing.

## CONFLICT OF INTEREST

The authors declare that they have no conflict of interests.

## ETHICAL STATEMENT

Ethical clearance and approval of the study were obtained from the Ethical Review Board of Metu University, Faculty of Public Health, and Medical Science. All study participants were informed about the confidentiality of the information and that they have a full right to participate or decline from participating in the study. Oral consent was obtained from every study subject and written consent was obtained from parents or guardians, for those less than 18 years.

## Supporting information


**DATA S1** Supporting information

## Data Availability

The authors confirm that the data supporting the findings of this study are available within the article and/or its supplementary materials.
