# Supplementary material for: Knowledge on cervical cancer, attitude toward its screening, and associated factors among reproductive age women in Metu Town, Ilu Aba Bor, South West Ethiopia, 2018: community‐based cross‐sectional study
Source: Cancer Rep (Hoboken). 2021 May 2;4(5):e1382. doi: 10.1002/cnr2.1382 (PMC8552000; doi:10.1002/cnr2.1382)
Supplement: Supplementary file 1 — DATA S1 Supporting information [file CNR2-4-e1382-s001.docx]

**Supplementary Materials**

**Ethical Considerations**

Ethical clearance and approval of the study were obtained from the Ethical Review Board of Metu University, Faculty of Public Health, and Medical science. All study participants were informed about the confidentiality of the information and that they have a full right to participate or decline from participating in the study. Oral consent was obtained from every study subject and written consent was obtained from parents or guardians, for those less than 18 years

**Acknowledgment**

We would like to extend our heart full gratitude to the Mettu University Department of Public Health for giving ethical clearance for this study. Our special thanks also go to the data collectors, supervisors, and all study participants for their time and willingness to participate in the study.

**Authors’ contribution**

KC Made substantial contributions to conception and design, acquisition of data, and analysis of this study and interpretation of data. DO and TS participated in data analysis and approved the final manuscript submitted TM Participated in data analysis and drafted the manuscript.

**Competing interest**

The authors declare that they have no conflict of interests

**Funding**

We (the authors) received no financial support for the research, authorship and or publication of the article.
